# Supplementary material for: Giant anomalous Hall effect from spin-chirality scattering in a chiral magnet
Source: Nat Commun. 2021 Jan 12;12:317. doi: 10.1038/s41467-020-20384-w (PMC7804464; doi:10.1038/s41467-020-20384-w)
Supplement: Supplementary file 1 — Supplementary Information [file 41467_2020_20384_MOESM1_ESM.pdf]

Supplementary Information for

Giant anomalous Hall effect from spin-chirality scattering in a chiral magnet

1. Formation of spin-hedgehog lattice in MnGe thin films

2. Electrical transport properties of the sample with the film thickness of 160 nm

3. Electric transport properties of the samples with the film thickness of 80 nm & 300 nm

4. Scaling relation between σ_{xy} and σ_{xx}

5. The analytical calculation for the thermal excitation of scalar-spin chirality (SSC)

6 Monte Carlo simulation of spin textures at high magnetic field

7. Discussion on the normal Hall effect

8. Chirality domain boundary

Supplementary Note 1: Formation of spin-hedgehog lattice in MnGe thin films.

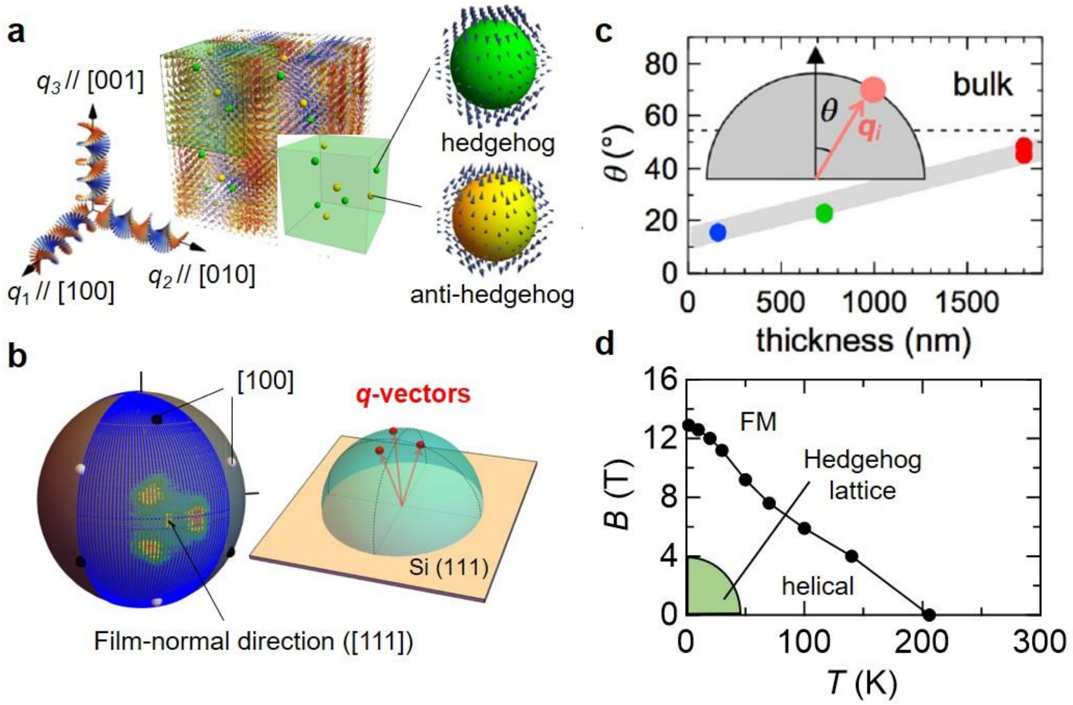

Supplementary Figure 1 | Formation of hedgehog lattice in MnGe thin films. a, The spin structures of hedgehog lattice in *bulk* MnGe, which is a three-dimensional array of hedgehogs and anti-hedgehogs. It can be described by the superposition of three helical modulation vectors (**q**-vectors) along the <100> crystal axes. **b,** The SANS intensity pattern of MnGe thin films with the film-thickness t = 160 nm, which is reproduced from Supplemetary Ref. 6. The three **q**-vectors are tilted to the film-normal direction [111], due the uniaxial anisotropy from the substrate. The consequence spin texture is the hedgehog lattice with a dilute density of hedgehogs and anti-hedgehogs. **c,** The effect of the film-thickness on the direction of the **q**-vectors revealed by the SANS experiment[S6]. θ is defined as the angle between the **q**-vectors and the film-normal direction, which is reduced with decreasing the film-thickness. This suggests the fact that the in-plane anisotropy of spin is enhanced in thinner films. **d,** The magnetic phase diagram of the MnGe thin film (t = 160 nm), which consists of the ferromagnetic (FM) phase, helical state, and hedgehog lattice state[S6].

Magnetic structures of topological spin crystals, such as skyrmion- and hedgehog-lattice states, can be described by the superposition of spin spirals with

propagation vectors **q**, *i.e.,* the multiple-q states[S1,S2]. Therefore, the small-angle neutron scattering (SANS) experiment has been one promising way to identify the topological spin texture, by detecting the **q**-vectors in reciprocal space. In bulk MnGe, three orthogonal **q**-vectors along <100> crystal axes have been observed by the SANS experiment[S2] as well as in the real-space by Lorentz TEM[S3]. The consequent spin texture becomes the dense array of spin hedgehogs and anti-hedgehogs connected by the skyrmion strings (*i.e.,* hedgehog lattice) (Supplementary Fig. 1**a**), which serve as the source (monopole) and sink (anti-monopole) of the emergent magnetic field, respectively[S4,S5]. Meanwhile, in the thin films of MnGe, the presence of uniaxial anisotropy modifies the mutual angles of the three **q**-vectors, resulting in the hedgehog lattice with dilute monopole/anti-monopole density[S6] (Supplementary Fig. 1**b**). In specific, the **q**-vectors are tilted to the film-normal direction from the <100> direction, showing the easy-plane anisotropy of spins in thin films (Supplementary Fig. 1**b**). Moreover, this in-plane anisotropy can be enhanced by decreasing the film-thickness (Supplementary Fig. 1**c**). The magnetic phase diagram of the MnGe thin film is shown in Supplementary Fig. 1**d**, where the hedgehog lattice is stabilized below 50 K and 4 T.

It should be noted here that recent STM study observed the multi domains with differently-oriented helical structures at the surface of a MnGe thin film[S7]. Since the internal magnetic structure has not been directly observed, we cannot know whether the multi-domain state may be maintained into the inside of the film or the multiple helical structures may interfere with each other and form a hedgehog lattice beneath the surface. Nevertheless, the observed multi-domain state also contains chains of hedgehogs and anti-hedgehogs at the disclination lines where helical structures with different propagation directions meet.

In either case, the main discussions on the SSC excitations and the consequent skew scatterings in the ferromagnetic region are unaffected by the incomplete understanding of ground magnetic state.

Supplementary Note 2: Electrical transport properties of the sample with the film thickness of 160 nm.

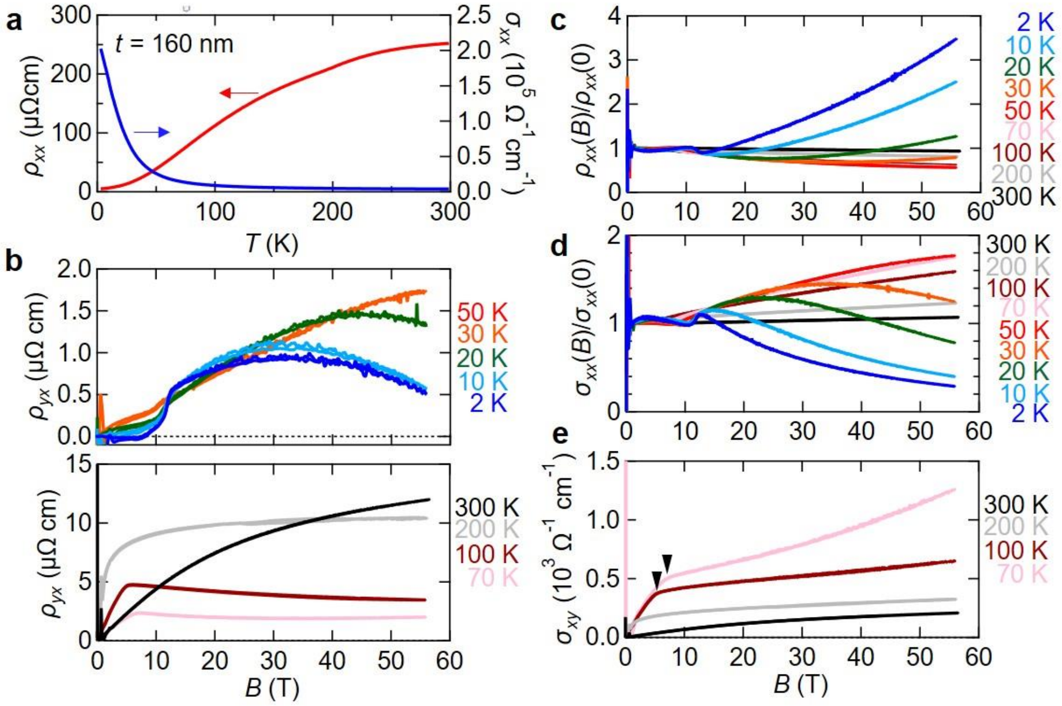

Supplementary Figure 2 | Electrical transport properties of a MnGe thin film (t = 160 nm). Complete data set for the sample (t = 160 nm). **a,** Temperature dependence of longitudinal resistivity (ρ_{xx}) and conductivity (σ_{xx}). **b,** Magnetic-field dependence of Hall resistivity (ρ_{yx}) at various temperatures. **c,** Normalized ρ_{xx} at various temperatures. **d,** Normalized σ_{xx} at various temperatures. **e,** Magnetic-field dependence of Hall conductivity (σ_{xy}) at various temperatures.

The temperature dependence of ρ_{xx} (and σ_{xx}) at zero magnetic field is shown in Supplementary Fig. 2**a**. The magnetic-field dependence ρ_{yx}, normalized ρ_{xx} and σ_{xx} are also shown in Supplementary Figs. 2**b-d**. The high-temperature ($T > 70$ K) data of σ_{xy} following the conventional intrinsic AHE curves are shown in Supplementary Fig. 2**e**, where the black triangles denote the ferromagnetic transition. The positive peak structures observed in ρ_{xx} or the negative peaks in σ_{xx} below the ferromagnetic transitions can be attributed to the fluctuations of the emergent magnetic field in the hedgehog lattice[S5]. The increase in ρ_{xx} (decrease in σ_{xx}) at high magnetic field originates from the cyclotron motion of electrons.

Supplementary Note 3: Electric transport properties of the samples with the film thickness of 80 nm & 300 nm.

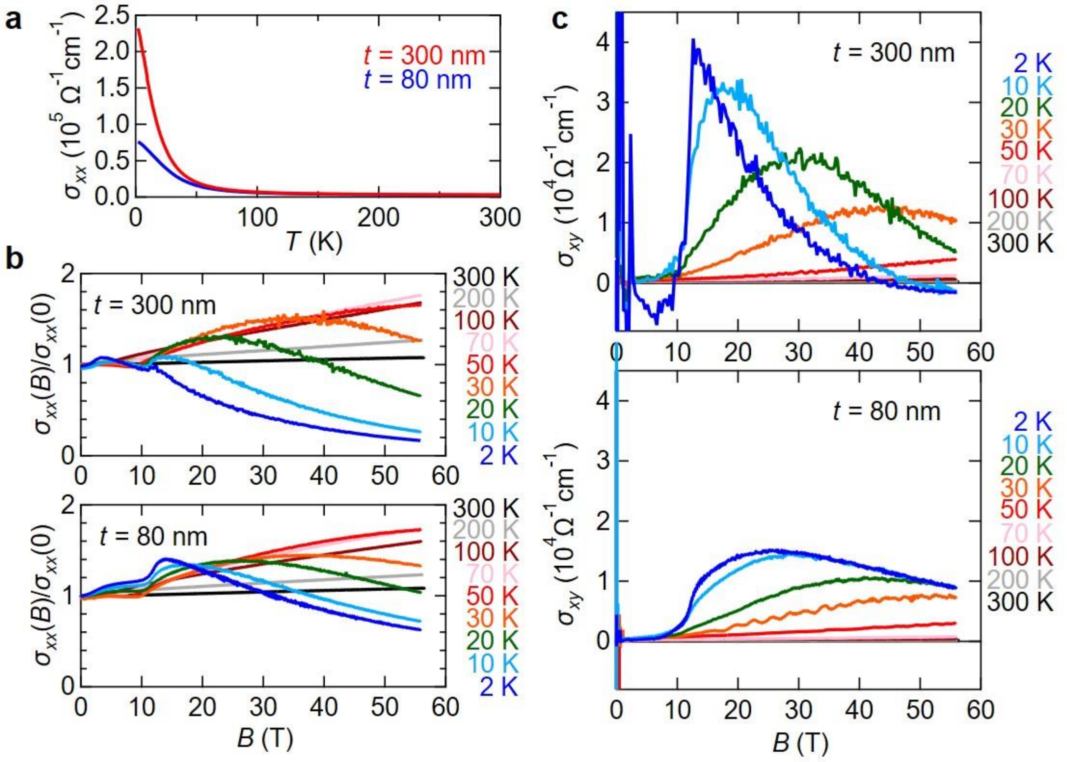

Supplementary Figure 3 | Electrical transport properties of MnGe thin films (t = 80 nm & 300 nm). Complete data set for the samples (t = 80 nm & 300 nm). **a,** Temperature dependence of longitudinal conductivity (σ_{xx}). **b,** Normalized σ_{xx} at various temperatures. **c,** Magnetic-field dependence of Hall conductivity (σ_{xy}) at various temperatures.

The temperature dependence of $\sigma_{xx} = 1/\rho_{xx}$ at zero magnetic field for the film thickness of 80 nm and 300 nm is shown in Supplementary Fig. 3**a**. The sample quality seems to become better (*i.e.,* the residual resistivity decreases) with increasing the film thickness in MnGe. The magnetic field dependence of σ_{xx} and σ_{xy} are summarized in Supplementary Figs. 3**b**,**c**. Importantly, the maximum value of the Hall angle (~ 20 %) is nearly independent of the film-thickness, suggesting the underlying common mechanisms (*i.e.*, spin-chirality skew-scattering).

We also note that the Hall angle is the intrinsic quantity, which is independent of the relaxation time or the sample quality in the framework of skew-scattering mechanism. Therefore, we have discussed the variation of the Hall angle against the film thickness as shown in Fig. 3 of the main text.

Supplementary Note 4: Scaling relation between σ_{xy} and σ_{xx}

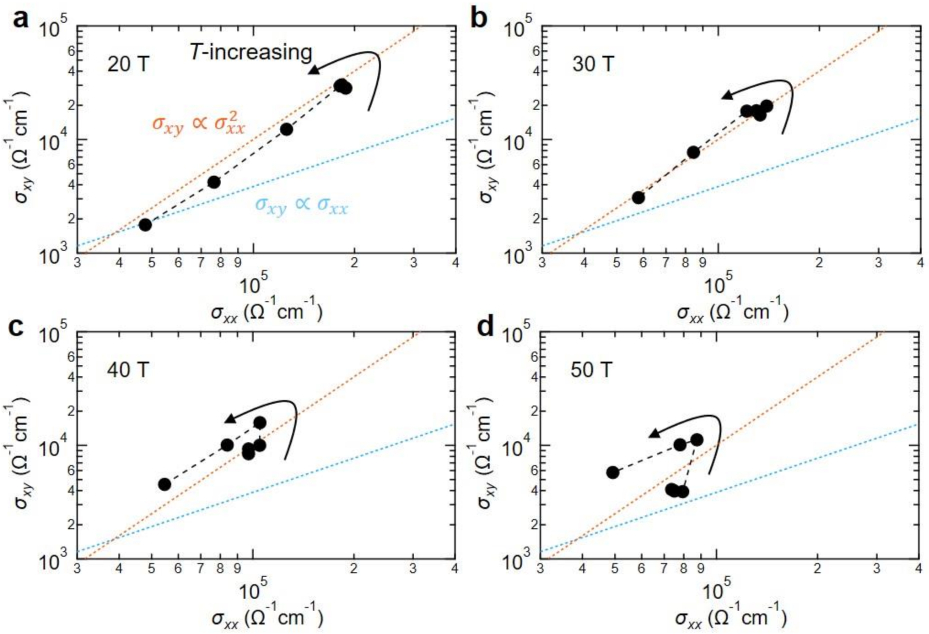

Supplementary Figure 4 | Full logarithmic plot of σ_{xy} versus σ_{xx} with varying temperature (*T* = 2-40 K) under fixed magnetic field (*t* = 160 nm, sample #1). The arrows denote the T-increasing direction while the orange/blue dashed lines are the guide for the scaling relations $\sigma_{xy} \propto \sigma_{xx}^2$ / $\sigma_{xy} \propto \sigma_{xx}$, respectively.

Since the normal Hall effect satisfies the relation $\sigma_{xy} = R_0 \sigma_{xx}^2 B$, where $R_0 = 1/ne$ is the normal Hall coefficient, the scaling relation of $\sigma_{xy} \propto \sigma_{xx}^2$ is expected under fixed B. As shown in Supplementary Fig.4, however, σ_{xy} plotted against σ_{xx} with varying temperature (T = 2-40 K) shows the complex behavior, and does not follow any kind of scaling relations (*i.e.*, $\sigma_{xy} \propto \sigma_{xx}^2$ for the normal Hall effect and $\sigma_{xy} \propto \sigma_{xx}$ for the conventional skew-scattering). We note that this result rather corroborates the spin-chirality skew-scatteirng mechanism, where the SSC excitation responds sensitively to the temperature and magnetic-field variation. If we take the data points where the Hall angle becomes the maximim at each temperature, the linear scaling relation $\sigma_{xy} \propto \sigma_{xx}$ holds true irrespective of the film-thickness or sample quality (see Fig. 2**a** in the main text). This is because the Hall angle reflects the magnitude of the SSC excitation (*i.e.* tilting angles of spins or density of monopole/anti-monopole exciation), and hence the data points used in Fig. 2**a** may share the same situations regarding the SSC excitation.

Supplementary Note 5: The analytical calculation for the thermal excitation of scalar-spin chirality (SSC).

We have reproduced the overall B-T profile of the SSC excitation by performing analytical calculations. For the low-temperature region, we describe the thermal fluctuation in terms of the low-energy excitations. At higher temperatures, we calculated the SSC using the high-temperature expansion of a chiral magnet. Based on these specific examples, we discuss the general behavior irrespective of model parameters.

- Low-temperature region

The low-energy excitations of the chiral magnets are spin-wave and the monopole/anti-monopole pair (skyrmion string) excitations. The existence of the low-energy skyrmion excitation owes to the fact that the spin hedgehog lattice phase exists in the vicinity of the field-forced ferromagnetic phase. The phase transition between the spin hedge hog lattice and the ferromagnetic orders are described by the condensation of skyrmion strings. Therefore, skyrmion strings exists as low-energy excitations even in the ferromagnetic state. In the continuum model, the skyrmion string excitations contribute to the scalar spin chirality while the spin wave contribution vanishes in the linear order. Therefore, we focus on the skyrmion string excitations in the rest of this section.

We assume a monopole/anti-monopole string with energy

$$E_{mn}(L) = 2E_0(1 \mp \delta) + [d(1 \mp \delta) + h]L, \tag{1}$$

where $E_0(1 \mp \delta)$ is the energy of the monopoles and anti-monopoles for skyrmion (+) and anti-skyrmion (-) strings, L is the length of the skyrmion string connecting the monopole and anti-monopole, h is the magnetic field, $d(1 \mp \delta)$ is the energy of skyrmion/anti-skyrmion string per unit length (we assume the string runs parallel to the magnetic field), $2d\delta$ is the energy difference between the skyrmion and anti-skyrmion strings. The energy difference reflects the difference of skyrmion and antiskyrmion spin textures. Microscopically, it is related to DM interaction and the magnetic field. We neglect the interaction between the monopoles because they are negligible if the monopole density is sufficiently small. Under these assumptions, the distribution function reads

$$Z = \sum_{n=0} \sum_{L_i=1} \exp\left[-\sum_{i=1}^{n} \frac{2E_0(1-\delta) + [d(1-\delta) + h - h_s]L_i}{T}\right]$$

$$\times \sum_{n=0} \sum_{L_i=1} \exp\left[-\sum_{i=1}^{n} \frac{2E_0(1+\delta) + [d(1+\delta) + h + h_s]L_i}{T}\right], \quad (2)$$

$$\sim \sum_{n=0}^{\infty} \left(\frac{e^{-\frac{2E_0(1-\delta)}{T}}}{1 - e^{-\frac{d(1-\delta)+h-h_s}{T}}} \right)^n \times \sum_{n=0}^{\infty} \left(\frac{e^{-\frac{2E_0(1+\delta)}{T}}}{1 - e^{-\frac{d(1+\delta)+h+h_s}{T}}} \right)^n, \quad (3)$$

$$= \frac{1}{1 - \left(\frac{e^{-\frac{2E_0(1-\delta)}{T}}}{1 - e^{-\frac{d(1-\delta)+h-h_s}{T}}} \right)} \frac{1}{1 - \left(\frac{e^{-\frac{2E_0(1+\delta)}{T}}}{1 - e^{-\frac{d(1+\delta)+h+h_s}{T}}} \right)}. \quad (4)$$

h_s is introduced for a technical purpose which will be clear in the following. We note that the transformation from the second to the third line holds only when

$$\left| \frac{e^{-\frac{2E_0(1\pm\delta)}{T}}}{1 - e^{-\frac{d(1\pm\delta)+h\pm h_s}{T}}} \right| < 1. \quad (5)$$

The chirality of the ferromagnetic phase is proportional to the density of the skyrmion strings. Therefore,

$$\chi \propto \frac{1}{Z} T \partial_{h_s} Z \bigg|_{h_s \to 0} = n_+ - n_-, \quad (6)$$

where

$$n_\pm = \frac{1}{\left(1 - e^{\frac{d(1\mp\delta)+h}{T}}\right)\left(1 + e^{\frac{2E_0(1\mp\delta)-d(1\mp\delta)-h}{T}} - e^{\frac{2E_0(1\mp\delta)}{T}}\right)}. \quad (7)$$

The result of the SSC as a function of temperature at various magnetic fields is shown in Supplementary Fig. 5**a**, which is consistent with the experimental result in the low-temperature limit shown in Fig. 2**c** of the main text. SSC is zero at zero temperature because it is the ferromagnetic state. At a finite temperature, the chirality becomes nonzero due to the imbalance of the population between skyrmion and anti-skyrmion strings.

- High-temperature region

The description based on the skyrmion/anti-skyrmion strings fails at a high temperature because of the weak spin correlation. Therefore, we employ the high-temperature expansion to discuss the B-T profile of the SSC in the high-temperature region $T \gg J$ (J is the energy scale of the ferromagnetic exchange interaction).

For this purpose, we here consider an fcc lattice model with weak Dzyaloshinskii-Moriya interaction (Supplementary Fig. 5**b**),

$$H = H_0 + H_1, \tag{8}$$

$$H_0 = -h\sum_i \hat{\mathbf{e}}_{[111]} \cdot \mathbf{S}_i^z - \frac{K}{2}\sum_i \left(\hat{\mathbf{e}}_{[111]} \cdot \mathbf{S}_i\right)^2, \tag{9}$$

$$H_1 = -J_1 \sum_{\langle i,j \rangle} \mathbf{S}_i \cdot \mathbf{S}_j - D \sum_{\langle i,j \rangle} \delta_{ij} \cdot \mathbf{S}_i \times \mathbf{S}_j + J_2 \sum_{\langle\langle i,j \rangle\rangle} \mathbf{S}_i \cdot \mathbf{S}_j. \tag{10}$$

Here, $\hat{\mathbf{e}}_{[111]}$ is the unit vector along the [111] axis. When $J_2 = K = 0$, this model is an fcc version of the effective model often used for MnSi[S8]. We also note that a related two-dimensional model has been studied by Leonov *et al*[S9]., which finds a skyrmion crystal phase at $T = 0$.

Below we focus on the SSC of the (111) plane. Using high-temperature expansion, we calculated the SSC in the paramagnetic/ferromagnetic phase of this model. The thermal average of the SSC for the nearest-neighbor triangles reads

$$\chi = < \mathbf{S}_1 \cdot (\mathbf{S}_2 \times \mathbf{S}_3) >, \tag{11}$$

where the site indices are assigned as in Supplementary Fig. 5**b**. In the high-temperature expansion, we expand the density matrix for $e^{-\beta H_1}$ with respect to β. To the second order, the thermal average reads

$$\chi \sim \frac{1}{Z}\mathrm{Tr}\left[e^{-\beta H_0}\left(1 - \beta H_1 + \frac{(\beta H_1)^2}{2}\right)\mathbf{S}_1 \cdot (\mathbf{S}_2 \times \mathbf{S}_3)\right]. \tag{12}$$

$$= \frac{3\sqrt{3}}{2}\beta^2 D^2 [\langle (S^z)^3 \rangle \langle (S^x)^2 \rangle \langle (S^y)^2 + (S^z)^2 \rangle \{\langle (S^x)^2 S^z \rangle \langle (S^y)^2 \rangle + \langle (S^x)^2 \rangle \langle (S^y)^2 S^z \rangle\}], \tag{S13}$$

Here, we take the spin axis so that S^z is parallel to the [111] axis.

We first focus on the $K = 0$ case. In this case,

$$\chi = \frac{3\sqrt{3}\beta^2 D^2}{2} F(\beta h), \tag{14}$$

$$F(x) = \frac{(1 - x\coth(x))[(2 + x^2)(9 + 2x^2) + x\coth(x)\{(x^2 + 18)x\coth(x) - 2(18 + 7x^2)\}]}{x^7}. \tag{15}$$

The result is the same for the downward triangles shown in Supplementary Fig. 5**b**.

The result of the SSC as a function of temperature at various magnetic fields is shown in Supplementary Fig. 5**c**, which is consistent with the experimental result of the high temperature shown in Fig. 2**c** of the main text. The SSC increases with increasing the magnetic field, which is related to the fact that the S^z terms increases with h, such as $\langle (S^z)^3 \rangle = \frac{\beta h}{5} + O(\beta^3 h^3)$. On the other hand, the terms related to S^x and S^y decreases with h, such as $\langle (S^x)^2 \rangle = \frac{1}{3} - \frac{\beta^2 h^2}{45} + O(\beta^3 h^3)$. Hence,

$$\chi = \frac{\beta^3 D^2 h}{6\sqrt{3}} + O(\beta^5). \tag{16}$$

We note that this is a general trend for the models with a triangular network of magnetic ions. Therefore, we expect a larger chirality at higher magnetic field, when $\beta D \ll 1$. The expected overall behavior of the SSC connecting the low-temperature and high-temperature regions is shown in Supplementary Fig. 5**d**, showing a peak structure at a finite temperature as observed in the experiment.

- Effect of easy-plane anisotropy

We consider the effect of anisotropy on the SSC excitation in this section. The single-ion anisotropy is incorporated in the thermal average term $\langle \cdots \rangle$ as

$$\langle \cdots \rangle \equiv \int \frac{d\theta d\emptyset \sin\theta}{Z} e^{\beta h \cos\theta + \frac{\beta K}{2}(\cos\theta)^2} (\cdots). \tag{17}$$

Therefore, the formula in Supplementary Eq. (13) holds with only difference in the definition of the thermal average. The leading-order effect from the anisotropy appears in the linear order of βK, which reads

$$\chi = \frac{3\sqrt{3}\beta^2 D^2}{2} [F(\beta h) + \beta K F_1(\beta h)], \tag{18}$$

$$\begin{aligned} F_1(x) = & -\left(\frac{342}{x^9} + \frac{294}{x^7} + \frac{73}{x^5} + \frac{4}{x^3}\right) \\ & + \left(\frac{972}{x^8} + \frac{569}{x^6} + \frac{78}{x^4}\right)\coth(x) - \left(\frac{864}{x^7} + \frac{273}{x^5} + \frac{5}{x^3}\right)(\coth(x))^2 \\ & + \left(\frac{180}{x^6} - \frac{15}{x^4}\right)(\coth(x))^3 + \left(\frac{54}{x^5} + \frac{3}{x^3}\right)(\coth(x))^4. \end{aligned} \tag{19}$$

Hence, the easy-plane anisotropy ($K > 0$) enhances the chirality. When $\beta h \ll 1$, the SSC

reads

$$\chi \sim \frac{\beta^3 D^2 h}{6\sqrt{3}} + \frac{\beta^4 D^2 h K}{75\sqrt{3}}. \tag{20}$$

Physically, the easy-plane anisotropy cause frustration between the magnetic field; the anisotropy tries to keep the spins in xy plane while the magnetic field prefers to point the spins in the perpendicular direction. The competition of anisotropy and magnetic field enhances the noncollinearlity. This observation is consistent with the results discussed in Fig. 3 in the main text, where the SSC excitation is enhanced in thinner films, even at low temperatures and high magnetic fields.

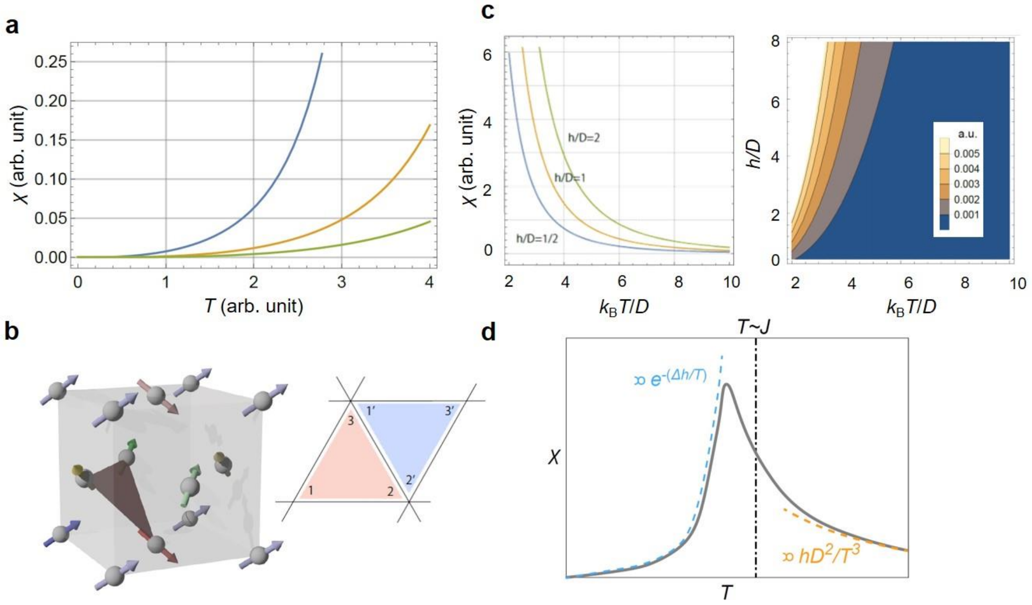

Supplementary Figure 5 | Analytical calculations on the scalar-spin chirality (SSC) excitation. a, Calculation of SSC in the low-temperature region by non-interacting skyrmion and anti-skyrmion strings. The calculation is for $E_0 = h - 1 + 0.5T, d = 1, \delta = 0.01$. The three lines are for different external magnetic fields: $h = 1$ (blue), $h = 1.5$ (orange), and $h = 2$ (green). **b,** A schematic figure of the fcc lattice model and the triangle we consider for the calculation of the SSC in the high-temperature series expansion. **c,** Calculations of the SSC in the high-temperature region. The three lines are for different external magnetic fields: $h/D = 1/2$ (blue), $h/D = 1$ (orange), and $h/D = 2$ (green). The contour map of the magnitude of SSC (χ) is also shown. **d,** Schematic figure of the SSC at a constant magnetic field, which connects the low-temperature and high-temperature regions. The peak structure of SSC resembles that of the Hall angle in MnGe.

Supplementary Note 6: Monte Carlo simulation of spin textures at high magnetic field

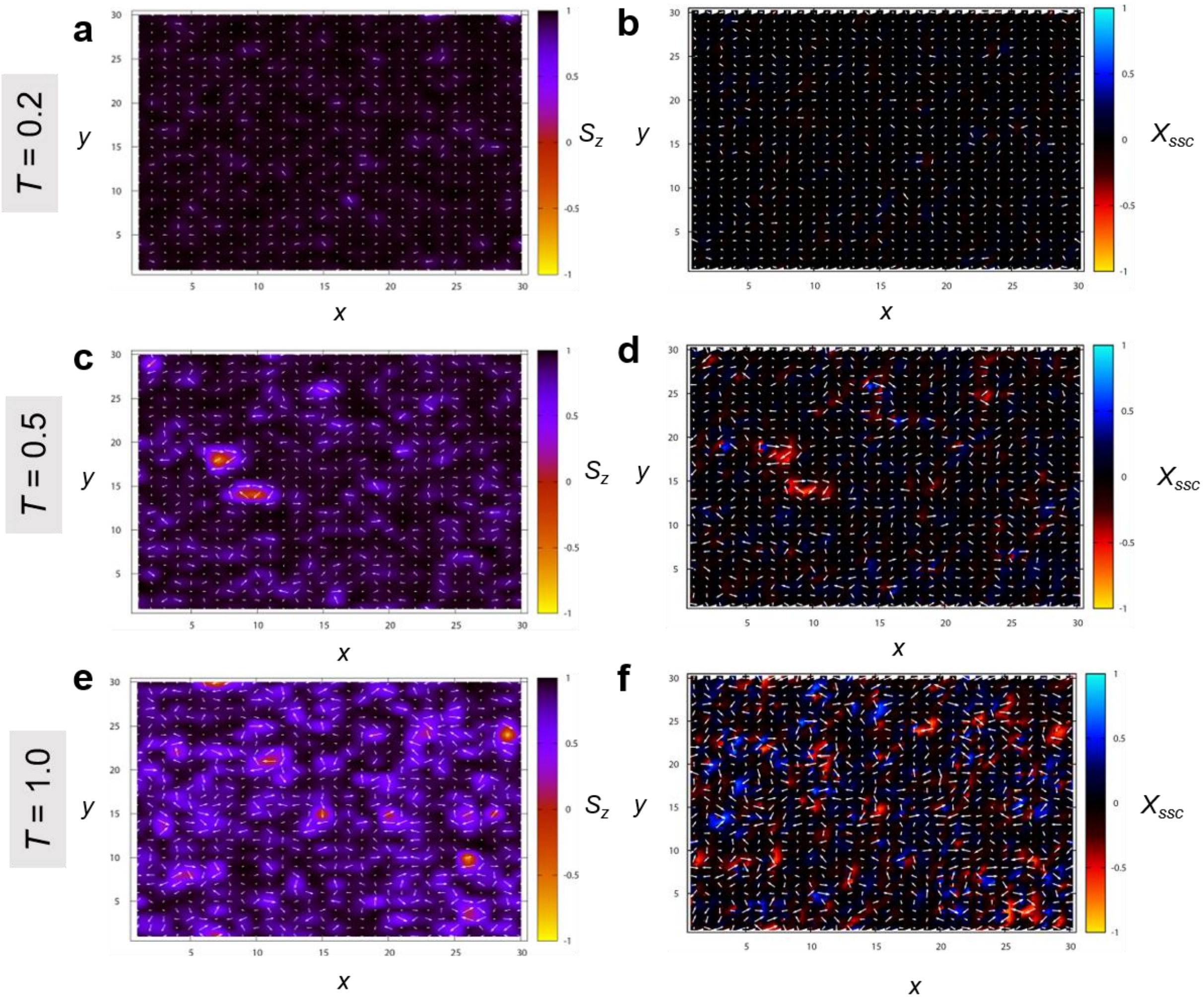

Supplementary Figure 6 | Monte Carlo simulation for chiral magnets at high magnetic field above the ferromagnetic transition. The snapshots of the spins at T = 0.2 (**a**,**b**), T = 0.5 (**c**,**d**), and T = 1.0 (**e**,**f**) at the magnetic field of B = 3.5. The magnetic ordering temperature (T_c) and the ferromagnetic transition temperature (B_c) for this model are T_c ~ 0.9 and B_c ~ 3.0 , respectively (see Supplementary Ref. S8 & S10 for detail). The color bars correspond to the z-component of spins (**a**,**c**,**e**) and the scalar-spin chirality (**b**,**d**,**e**), respectively.

We performed a Monte Carlo simulation for chiral magnets to show that the topological excitations survive at high magnetic fields well above the FM transition. To see how the topological excitations are generated in a high magnetic field in terms of a

tractable model, we consider a two-dimensional model for chiral magnet[S11].

$$H = -J\sum_{r} \vec{S}(\vec{r}) \cdot \vec{S}(\vec{r}+\hat{x}) + \vec{S}(\vec{r}) \cdot \vec{S}(\vec{r}+\hat{y})$$
$$- D\sum_{r} \hat{x} \cdot \vec{S}(\vec{r}) \times \vec{S}(\vec{r}+\hat{x}) + \hat{y} \cdot \vec{S}(\vec{r}) \times \vec{S}(\vec{r}+\hat{y}) - B\sum_{r} S^{z}(\vec{r}) . (21)$$

We take $D/J = \sqrt{6}$ and $B/J = 3.5$ in this manuscript. This model shows three phases under the magnetic field: helical phase in the low field, skyrmion crystal phase in the intermediate field, and the field-forced FM phase in the high field[S8]. The transition from the skyrmion crystal phase to the FM phase occurs at *B/J* ~ 3 for the parameters mentioned above[S10].

Supplementary Figs. 6 shows the snapshots taken from the simulation at *B* =3 .5. As shown in Supplementary Figs. 6**a,b**, the SSC excitation is suppressed at the low temperatures, *T/J* = 0.2. Upon increasing the temperature, the SSC excitation (or skyrmionic excitation) emerges as in the snapshots for *T/J* = 0.5 (Supplementary Figs. 6**c,d**). The density of skyrmonic excitations increases monotonically with increasing the temperature. However, as the temperature increases, the skyrmionic excitations with the opposite chirality (or anti-skyrmionic excitation) also appear, reducing the total SSC density (Supplementary Figs. 6**e,f**). These results are consistent with our experimental observation, where the anomalous Hall effect shows a maximum at a finite temperature under a fixed magnetic field (Fig. 2**c**).

Supplementary Note 7: Discussion on the normal Hall effect.

- **Fitting σ_{xy} with single-carrier Drude model**

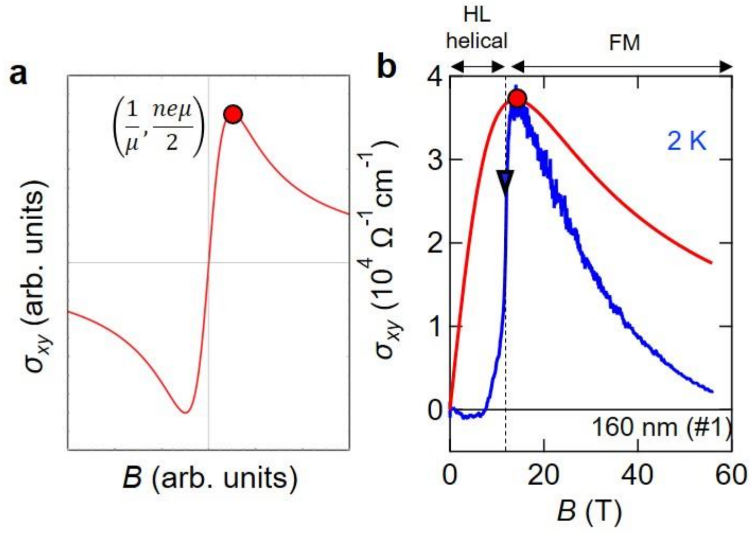

Supplementary Figure 7 | Fitting the Hall conductivity σ_{xy} by single-carrier Drude model. a, Schematics of the "dispersive resonance" profile of σ_{xy} following a Drude model. The peak position is determined by the inverse of mobility μ. **b,** Fitting of σ_{xy} [2 K, 160nm (sample#1)] by a single-carrier Drude model (red line), while employing carrier density and mobility estimated from the peak position (red solid circle). The black triangle denotes the ferromagnetic (FM) transition.

σ_{xy} with a sharp peak structure ("dispersive-resonance" profile), can arise from the normal Hall effect when the carrier mobility μ is sufficiently large, as typically observed in Dirac or Weyl semimetals[S12]. Here, the profile of σ_{xy} follows the Drude model [$\sigma_{xy} = \mu ne \cdot \mu B/(1 + (\mu B)^2)$], where the peak position (magnetic field) is determined by the inverse of μ (Supplementary Fig. 7**a**). In the case of MnGe, one possibility is that the ferromagnetic phase transition entails the emergence of a high-mobility carrier pocket, such as the magnetic Weyl points. Although MnGe is a multi-band system with a relatively large carrier density, the existence of one high-mobility pocket might dominate the low-field transport, resulting in the "dispersive-resonance" profile. Therefore, we first tried to fit σ_{xy} with a single-carrier Drude model while fixing the peak position, assuming that the peak structure were produced by such high-mobility carriers. As shown in Supplementary Fig. 7**b**, the observed σ_{xy} largely deviates from the

Drude curve.

- **Fitting σ_{xy} with two-carrier Drude model**

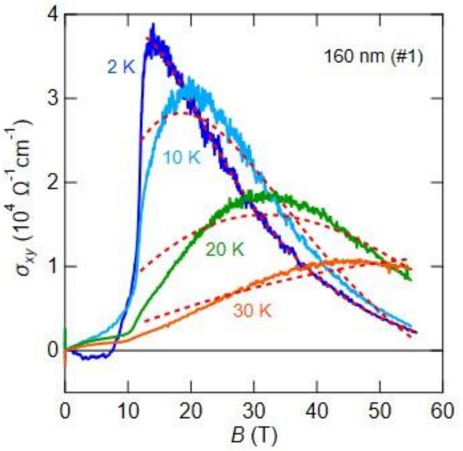

Supplementary Figure 8 | Fitting the Hall conductivity σ_{xy} by two-carrier Drude model. The red dashed line shows the result of fitting.

We also tried fitting σ_{xy} with two-carrier Drude model [$\sigma_{xy} = \mu_h n_h e \cdot \mu_h B/(1+(\mu_h B)^2) + \mu_e n_e e \cdot \mu_e B/(1+(\mu_e B)^2)$], assuming the high-mobility hole pocket and the low-mobility electron pocket, as shown by the red dashed lines in Supplementary Fig. 8. The fitting is performed above the ferromagnetic transition, and obtained parameters are $n_h = 9.4 \times 10^{20}$ cm^{-3}, $\mu_h = 690$ cm^{2}V^{-1}s^{-1}, $n_e = 1.2 \times 10^{21}$ cm^{-3}, $\mu_e = 250$ cm^{2}V^{-1}s^{-1} at $T = 2$ K. Multi-carrier Drude model, in general, may reproduce any kind of σ_{xy} as we further increase the number of carriers or fitting parameters. Hence we cannot completely exclude the possibility of the normal Hall effect, and further experiments such as the direct observation of electronic structure would be necessary to discuss the possibility of magnetic Weyl points. However, we assume that the normal Hall effect is less likely to dominate the observed large response, based on the discussions regarding the comparison between bulk crystals of MnGe as shown below.

- **Comparison of Hall conductivity between thin film, single crystal, and poly crystal**

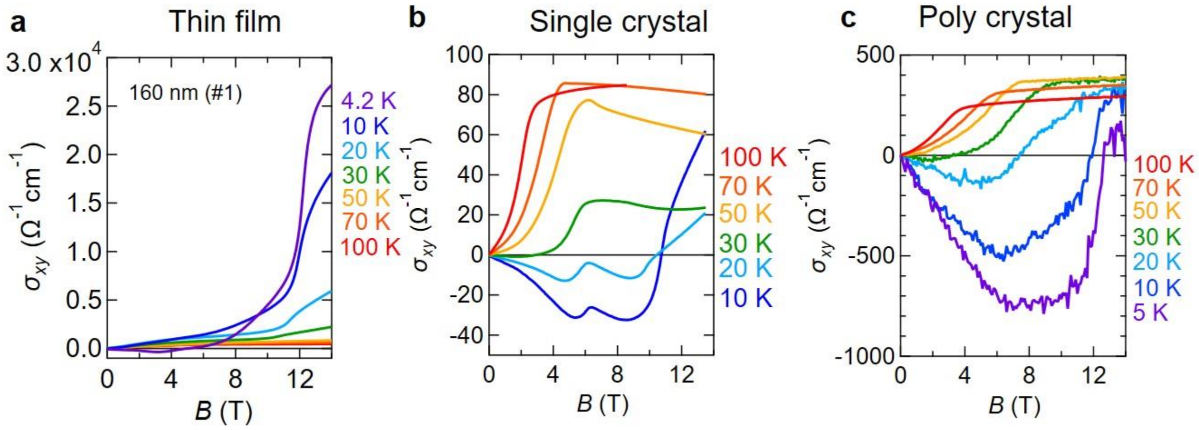

Supplementary Figure 9 | Hall conductivity at various temperatures for thin film, single crytstal, and poly crystal of MnGe. The enhancement of Hall conductivity in the ferromagnetic region is not observed in bulk poly crystal of MnGe.

The large enhancement of the Hall response at low temperature is observed only in the thin-films and bulk single-crystals[S13], but NOT in bulk poly-crystals of MnGe[S4] (Supplementary Fig. 9). Because the value of σ_{xx}, which reflects the carrier density or mobility, is almost identical between thin-films ($\sigma_{xx} = 2.0 \times 10^5$ Ω^{-1}cm^{-1} at 2 K) and poly-crystals ($\sigma_{xx} = 1.6 \times 10^5$ Ω^{-1}cm^{-1} at 2 K), this result suggests that the observed large Hall response may NOT be dominated by the normal Hall effect.

In terms of the spin-chirality skew-scattering mechanism, we speculate that the presence of random crystal domains in poly-crystals may weaken the total SSC excitation.

Supplementary Note 8: Chirality domain boundary

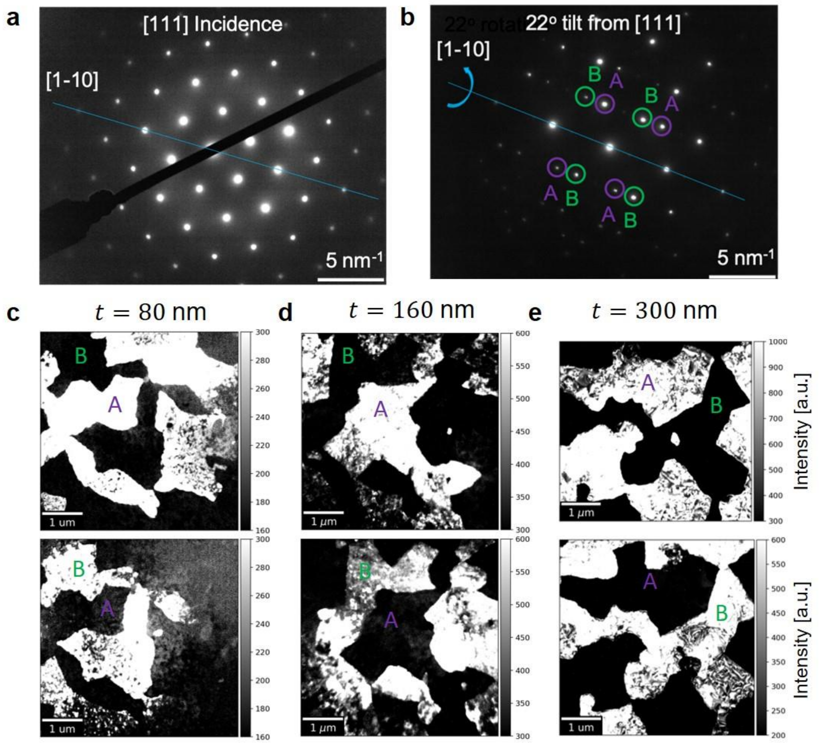

Supplementary Figure 10 | Dark-field TEM measurement on MnGe thin films. a, Electron diffraction pattern with [111] incidence. The MnGe domains of interest are found by rotating around the $[11\overline{2}]$ axis by approximately 22°. **b,** The diffraction pattern observed at such a tilt, with the peaks corresponding to the two chiral domains, labeled A and B. **c-e,** Dark-field TEM images of film-thickness t = 80 nm (**c**), 160 nm (**d**), and 300 nm (**e**), using diffraction peaks A (upper panels) and B (lower panels), respectively. Bright parts correspond to the domains A and B, respectively.

The growth of MnGe layer on the Si(111) substrate leads to the formation of enantiomorphic twins in MnGe thin films, which was investigated by the dark-field TEM measurement. As shown in Supplementary Figs. 10**c-e**, we observed a typical domain size of ~2-3 μm, regardless of the film thickness. These lattice-chirality domain boundaries may serve as the pinning potential for the thermal excitation of the SSC, however, we conclude that they may have small effects, if any, in the observed Hall effects, since the Hall response dramatically changes with the film-thickness while the domain sizes remain nearly constant.

Supplementary References

S1. Mühlbauer, S. *et al*. Skyrmion lattice in a chiral magnet. *Science*, **323**, 915 (2009).

S2. Kanazawa, N. *et al*. Possible skyrmion-lattice ground state in the *B*20 chiral-lattice magnet MnGe as seen via small-angle neutron scattering. *Phys. Rev. B* **86**, 134425 (2012).

S3. Tanigaki, T. *et al*. Real-space observation of short-period cubic lattice of skyrmions in MnGe. *Nano Lett.* **15**, 5438-5442 (2015).

S4. Kanazawa, N. *et al*. Large topological Hall effect in a short-period helimagnet MnGe. *Phys. Rev. Lett.* **106**, 156603 (2011).

S5. Kanazawa, N. *et al*. Critical phenomena of emergent magnetic monopoles in a chiral magnet. *Nat. Commun.* **7**, 11622 (2016).

S6. Kanazawa, N *et al*. Topological spin-hedgehog crystals of a chiral magnet as engineered with magnetic anisotropy. *Phys. Rev. B* **96**, 220414(R) (2017).

S7. Repicky, J *et al*. Atomic scale visualization of topological spin textures in the chiral magnet MnGe. arXiv:2008.00886 (2020).

S8. Yu, X. Z. *et al*. Real-space observation of a two-dimensional skyrmion crystal. *Nature* **465**, 901-904 (2010).

S9. Leonov, A. O. & Mostovoy, M. Edge states and skyrmion dynamics in nanostripes of frustrated magnets. *Nat. Commun.* **8**, 14394 (2017).

S10. Ishizuka, H. & Nagaosa, N. Spin chirality induced skew scattering and anomalous Hall effect in chiral magnets. *Sci. Adv.* **4**, eaap9962 (2018).

S11. Landau, L. D., Lifshitz, E. M. & Pitaevskii, L. P. *Electrodynamics of Continuous Media* (Elsevier, 2008).

S12. Liang, T. *et al*. Ultrahigh mobility and giant magnetoresistance in the Dirac semimetal Cd_3As_2. *Nat. Mater.* **14**, 280-284 (2015).

S13. Kanazawa. N, *et al*. Direct observation of the statics and dynamics of emergent magnetic monopoles in a chiral magnet. *Phys. Rev. Lett.* **125**, 137202 (2020)
